# Direct versus indirect actions of ghrelin on hypothalamic NPY neurons

**DOI:** 10.1371/journal.pone.0184261

**Published:** 2017-09-06

**Authors:** Hiroshi Hashiguchi, Zhenyu Sheng, Vanessa Routh, Volodymyr Gerzanich, J. Marc Simard, Joseph Bryan

**Affiliations:** 1 Department of Diabetes and Endocrinology, Kagoshima University Graduate School of Medicine and Dental Science, Kagoshima, Kagoshima, Japan; 2 Department of Pharmacology, Physiology and Neuroscience, Rutgers The State University of New Jersey, Newark, New Jersey, United States of America; 3 Department of Neurosurgery, University of Maryland School of Medicine, Baltimore, Maryland, United States of America; 4 Pacific Northwest Diabetes Research Institute, Seattle, Washington, United States of America; Cinvestav-IPN, MEXICO

## Abstract

**Objectives:**

Assess direct versus indirect action(s) of ghrelin on hypothalamic NPY neurons.

**Materials and methods:**

Electrophysiology was used to measure ion channel activity in NPY-GFP neurons in slice preparations. Ca^2+^ imaging was used to monitor ghrelin activation of isolated NPY GFP-labeled neurons. Immunohistochemistry was used to localize Trpm4, SUR1 and Kir6.2 in the hypothalamus.

**Results:**

Acylated ghrelin depolarized the membrane potential (MP) of NPY-GFP neurons in brain slices. Depolarization resulted from a decreased input resistance (IR) in ~70% of neurons (15/22) or an increased IR in the remainder (7/22), consistent with the opening or closing of ion channels, respectively. Although tetrodotoxin (TTX) blockade of presynaptic action potentials reduced ghrelin-induced changes in MP and IR, ghrelin still significantly depolarized the MP and decreased IR in TTX-treated neurons, suggesting that ghrelin directly opens cation channel(s) in NPY neurons. In isolated NPY-GFP neurons, ghrelin produced a sustained rise of [Ca^2+^]_c_, with an EC_50_ ~110 pM. Pharmacologic studies confirmed that the direct action of ghrelin was through occupation of the growth hormone secretagogue receptor, GHS-R, and demonstrated the importance of the adenylate cyclase/cAMP/protein kinase A (PKA) and phospholipase C/inositol triphosphate (PLC/IP_3_) pathways as activators of 5' AMP-activated protein kinase (AMPK). Activation of isolated neurons was not affected by CNQX or TTX, but reducing [Na^+^]_o_ suppressed activation, suggesting a role for Na^+^-permeable cation channels. SUR1 and two channel partners, Kir6.2 and Trpm4, were identified immunologically in NPY-GFP neurons *in situ*. The actions of SUR1 and Trpm4 modulators were informative: like ghrelin, diazoxide, a SUR1 agonist, elevated [Ca^2+^]_c_ and glibenclamide, a SUR1 antagonist, partially suppressed ghrelin action, while 9-phenanthrol and flufenamic acid, selective Trpm4 antagonists, blocked ghrelin actions on isolated neurons. Ghrelin activation was unaffected by nifedipine and ω-conotoxin, inhibitors of L- and N-type Ca^2+^ channels, respectively, while Ni^2+^, mibefradil, and TTA-P2 completely or partially inhibited ghrelin action, implicating T-type Ca^2+^ channels. Activation was also sensitive to a spider toxin, SNX-482, at concentrations selective for R-type Ca^2+^ channels. Nanomolar concentrations of GABA markedly inhibited ghrelin-activation of isolated NPY-GFP neurons, consistent with chronic suppression of ghrelin action *in vivo*.

**Conclusions:**

NPY neurons express all the molecular machinery needed to respond directly to ghrelin. Consistent with recent studies, ghrelin stimulates presynaptic inputs that activate NPY-GFP neurons *in situ*. Ghrelin can also directly activate a depolarizing conductance. Results with isolated NPY-GFP neurons suggest the ghrelin-activated, depolarizing current is a Na^+^ conductance with the pharmacologic properties of SUR1/Trpm4 non-selective cation channels. In the isolated neuron model, the opening of SUR1/Trpm4 channels activates T- and SNX482-sensitive R-type voltage dependent Ca^2+^ channels, which could contribute to NPY neuronal activity *in situ*.

## Introduction

Ghrelin, the peptide ligand of the growth hormone secretagogue receptor (GHS-R) [1,2], plays multiple roles in feeding behavior and energy balance [3] via central and peripheral actions [4]. GHS-R is a G-protein-coupled receptor present in high density in the hypothalamus and pituitary gland [1,5,6,7,8] that couples with Gs and Gq proteins to stimulate adenylate cyclase/cAMP/PKA and PLC/IP_3_ pathways, respectively [9]. The long form of the receptor, GHS-R type 1a, is strongly expressed in the arcuate nucleus (ARC), ventromedial nucleus (VMN), and paraventricular nucleus (PVN) in the hypothalamus [5,10,11], especially in NPY and GHRH neurons [12,13,14]. The presence of GHS-R has also been reported in extrahypothalamic areas of the CNS including the cerebral cortex, dentate gyrus, CA2 and CA3 regions of the hippocampus, parafascicular thalamic region, substantia nigra, ventral tegmental area, raphe nuclei, nodose ganglion and dorsal vagal complex (reviewed in [3,4]). Neuropeptide Y (NPY) and Agouti-related peptide (AgRP) are potent stimulators of food intake (reviewed in [15]). These neuropeptides are synthesized in the arcuate nucleus by NPY/AgRP neurons, which are essential players in the control of feeding behavior and body weight [16,17]. Ghrelin’s actions on food intake are mediated by NPY and AgRP, thus ghrelin exerts its orexigenic action(s) through activation of NPY/AgRP neurons [18,19,20]. While NPY neurons receive multiple synaptic inputs from neighboring cells [21], they also have endogenous ghrelin receptors, and thus the relative functions of presynaptic input versus direct ghrelin action are of interest.

Ghrelin is reported to activate NPY neurons via multiple mechanisms, all of which are functional in isolated neurons including the adenylate cyclase/cAMP/PKA, PLC/IP_3_ and AMPK pathways. The ion channels modulated by these pathways are not fully understood. A recent study reported that ghrelin *hyperpolarizes* neurons in the nodose ganglion by activating SUR1/Kir6.2-type K_ATP_ channels [22], and previous reports described SUR1/Kir6.2-type K_ATP_ channels in AgRP/NPY neurons *in situ* [23,24,25].

Here, to assess the direct and indirect effects of ghrelin, electrophysiological recordings were done on NPY-GFP neurons in brain slices from NPY-GFP mice [26] and Ca^2+^-imaging was carried out on isolated NPY-GFP neurons. We found that, *in situ*, ghrelin *depolarized* the membrane potential (MP) of NPY-GFP neurons by either increasing or decreasing the input resistance (IR) implicating channel closings and openings, respectively. Tetrodotoxin (TTX) attenuated the action of ghrelin on both MP and IR, consistent with blockade of ghrelin-mediated presynaptic inputs, but in TTX-treated VMH slices ghrelin still significantly depolarized the MP and decreased IR, implying direct activation of ion channels in NPY neurons.

Stimulation of isolated NPY-GFP neurons by diazoxide, a commonly used SUR1 K_ATP_ channel agonist, paradoxically produced neuronal depolarization and sustained elevation of [Ca^2+^]_c_, while glibenclamide, a SUR1 antagonist, blunted the actions of ghrelin. SUR1 partners with Kir6.2 to assemble K_ATP_ channels whose activation will hyperpolarize cells, but SUR1 also pairs with Trpm4 to form SUR1/Trpm4 non-selective cation channels, whose openings would depolarize neurons to ~0 mV [27]. While SUR1, Kir6.2 and Trpm4 were identified immunologically in arcuate NPY neurons, the results with SUR1 modulators are consistent with ghrelin stimulating SUR1/Trpm4 channels [27], not neuroendocrine-type K_ATP_ channels. These results imply that ghrelin, in addition to modulating presynaptic inputs, may activate non-selective cation channels directly in NPY neurons and contribute to activation of T- and R-type voltage-dependent Ca^2+^ channels.

## Materials and methods

### Animals

NPY-GFP mice were obtained from the Jackson Laboratory (Bar Harbor, ME). NPY-GFP mice were maintained on a 12-hr light/dark cycle and given free access to food and water. All animal protocols were approved by the Institutional Animal Care and Use Committee at the Pacific Northwest Diabetes Research Institute (Seattle, WA) and the Rutgers New Jersey Medical School (Newark, NJ).

### Isolation of NPY-GFP neurons from the ventromedial hypothalamus

Brain slices containing the ventromedial hypothalamus (VMH) were prepared; single neurons were isolated as described [28,29,30] with modifications. Briefly, three-to-four-week-old male mice were anesthetized by an intraperitoneal injection of sodium pentobarbital (50-100mg/kg) then transcardially perfused with an ice-cold oxygenated (95% O_2_-5% CO_2_) solution composed of (mM):2.5 KCl, 1.25 NaH_2_PO_4_, 7 MgCl_2_, 0.5 CaCl_2_, 28 NaHCO_3_, 7 glucose, 1 ascorbate, and 3 pyruvate, pH 7.4, with osmolarity adjusted to ~300 mosmol/L H_2_O. Brains were removed and placed in a slushy ice cold perfusion solution; coronal hypothalamic sections (300μm) containing the VMH were cut using a vibroslicer (Vibroslice NVSL, Sarasota, FL). Slices were transferred to a dissection dish containing HibernateA/B27 medium plus 2.5mM glucose at 4°C. The VMH was dissected and digested with papain (final concentration 15–20 U/ml in HibernateA) for 25–30 minutes in a 37°C water bath rotating at 100 rpm. The tissue was then rinsed with HibernateA/B27 and gently triturated. The resulting cell suspension was centrifuged and the pellet re-suspended in astrocyte conditioned medium (ACM) containing 2.5 mM glucose [31,32,33]. Neurons were plated on coverslips in ACM and used within 10 hr.

### Electrophysiology

After anesthetization, 4–6 week old male NPY-GFP mice were transcardially perfused with ice-cold oxygenated (95% O_2_/5% CO_2_) perfusion solution: 2.5 mM KCl, 7 mM MgCl_2_, 1.25 mM NaH_2_PO_4_, 28 mM NaHCO_2_, 0.5 mM CaCl_2_, 7 mM glucose, 1 mM ascorbate, 3 mM pyruvate; osmolarity was adjusted to -300 mOsm with sucrose; pH 7.4. Coronal sections (300 μm) through the ARC were made on a vibratome (Leica Instruments) as described [34]. Prior to recording, the brain slices were incubated at room temperature for 1 h in oxygenated artificial cerebrospinal fluid (aCSF; 126 mM NaCl, 1.9 mM KCl, 1.2 mM KH_2_PO_4_, 26 mM NaHCO_3_, 2.5 mM glucose, 1.3 mM MgCl_2_, and 2.4 mM CaCl_2_; osmolarity was adjusted to -300 mOsm with sucrose; pH 7.4).

The standard whole cell recording configuration was established in visually identified NPY-GFP neurons using a MultiClamp 700A amplifier (Axon Instruments, Foster City, CA). Data were analyzed using pClamp9 software. During recording, brain slices were perfused at 6 ml/min with oxygenated aCSF. Borosilicate pipettes (4.0–4.5 MΩ) were filled with an intracellular solution containing: 128 mM K-gluconate, 10 mM KCl, 10 mM KOH, 10 mM HEPES, 4 mM MgCl_2_, 0.05 mM CaCl_2_, 0.5 mM EGTA, 2 mM Na_2_ATP and 0.4 mM Na_2_GTP; pH 7.2. Osmolarity was adjusted to 290–300 mOsm with sucrose. Neurons with access resistance more than 35 MΩ during the recording were not used. Junction potential was calculated and corrected offline after the recordings. Input resistance was calculated using Ohm's Law (Resistance (R) = Voltage (V)/Current (I)), from the membrane voltage change in response to hyperpolarizing current pulses of −10 or −20 pA. Membrane potential and IR were calculated during the last one minute of each control period or treatment application.

### Measurements of intracellular Ca^2+^ ([Ca^2+^]_c_) in NPY neurons

[Ca^2+^]_c_ was measured by ratiometric fura-2 microfluorometry within 10 hours of neuron isolation. Following a 30-minute loading with 2 μM fura-2AM at 37°C, coverslips with cells were placed in a custom-made flow chamber with a volume of 1.5 ml. An extracellular solution (in mM: 135 NaCl, 5 KCl, 1 CaCl_2_, 1 MgCl_2_, 10 HEPES, and 2.5 glucose, pH 7.4, 37°C) was pumped through the chamber at a rate of 1 ml/minute. Solutions were switched manually with a switch time of a few seconds, the estimated time for total solution exchange time in the chamber was ~2minutes. The GFP signal was used to identify NPY neurons. Measurements were carried out using a Leica DM6000B microscope with a 20X objective. Excitation was at 340 and 380 nm; emission was recorded at 510 nm at 8 second intervals. The data are expressed as Relative [Ca^2+^]_c_ defined as the ratio of the emitted light at 510 nm following sequential excitation at 340 vs 380 nm. Ratio images were analyzed using the Leica Application Suite Advanced Fluorescence software (Leica Microsystems, Wetzlar, Germany).

### Immunofluorescence labeling

NPY-GFP mice, 7–8 weeks old, were euthanized and transcardially perfused with 4% paraformaldehyde (PFA) in phosphate buffered saline (PBS). The brains were harvested, immersion fixed overnight in 4% PFA/PBS, then transferred to 30% sucrose/PBS. Coronal cryosections (10 μm) on glass slides were blocked (2% donkey serum + 0.2% Triton X-100 for 1 hour at room temperature), then incubated overnight at 4°C with primary antibodies (see below). After several rinses in phosphate-buffered saline, the slides were incubated for 1 hour with fluorescent-labeled species-appropriate secondary antibodies (1:500; Alexa Fluor 488 and Alexa Fluor 555; Invitrogen, Molecular Probes, Eugene, OR, USA) at room temperature. Omission of primary antibody was used as a negative control. The sections were coverslipped using polar mounting medium containing antifade reagent and 4′,6-diamidino-2-phenylindole (DAPI; Invitrogen, Eugene, OR, USA) and were examined using epifluorescence microscopy (Nikon Eclipse 90i; Nikon Instruments Inc., Melville, NY, USA). The following primary antibodies were used: goat anti-SUR1 (1:200, Santa Cruz Biotechnology, Santa Cruz, CA); rabbit anti-Kir6.2 antibody (1:200, Santa Cruz); chicken anti-Trpm4 antibody (1:200; custom antibody, as described in Woo *et al*. [27]).

### Solutions and chemicals

Acylated ghrelin was obtained from Phenix Pharmaceuticals (Burlingame, CA). Compound-C was obtained from Calbiochem (La Jolla, CA). SNX-482, AICAR, STO-609, ω-Conotoxin, GABA and [D-Lys^3^]-GHRP-6 were obtained from Tocris (Ellsville, MO). Sodium gluconate was obtained from Spectrum (Garden, CA). Choline chloride and flufenamic acid were obtained from Acrōs (Fair Lawn, NJ). Hibernate A was obtained from Brainbits (Carlsbad, CA). Neurobasal A, B27, Fura-2 and Alexa fluor 546 conjugated secondary antibodies were obtained from Invitrogen (Springfield, IL). Papain was obtained from Worthington (Lakewood, NJ). The T-type channel inhibitor, TTA-P2, was generously provided by Dr. Victor N. Uebele (Merck Research Labs, West Point, PA). All other chemicals were from Sigma Chemical Corp (Saint Louis, MO). All agents were dissolved in DMSO or distilled water; DMSO was diluted in extracellular solution at ≥1:1,000.

### Data presentation and statistical analysis

For Ca^2+^ imaging experiments the signals from 5–20 neurons were averaged and the mean values plotted as traces in the figures. All experiments were repeated at least three times using independent neuron preparations. Electrophysiological measurements were done on 22 GFP-labeled neurons without and 9 neurons with TTX present.

Statistical calculations were done and plots produced using Origin 2017 (OriginLab Corp, Northampton, MA). Paired two-tailed t-tests were done using GraphPad Instat 3.10 (GraphPad Software, San Diego California USA).

## Results

### Ghrelin activates NPY-GFP neurons directly and indirectly *in situ*

**Fig 1:**
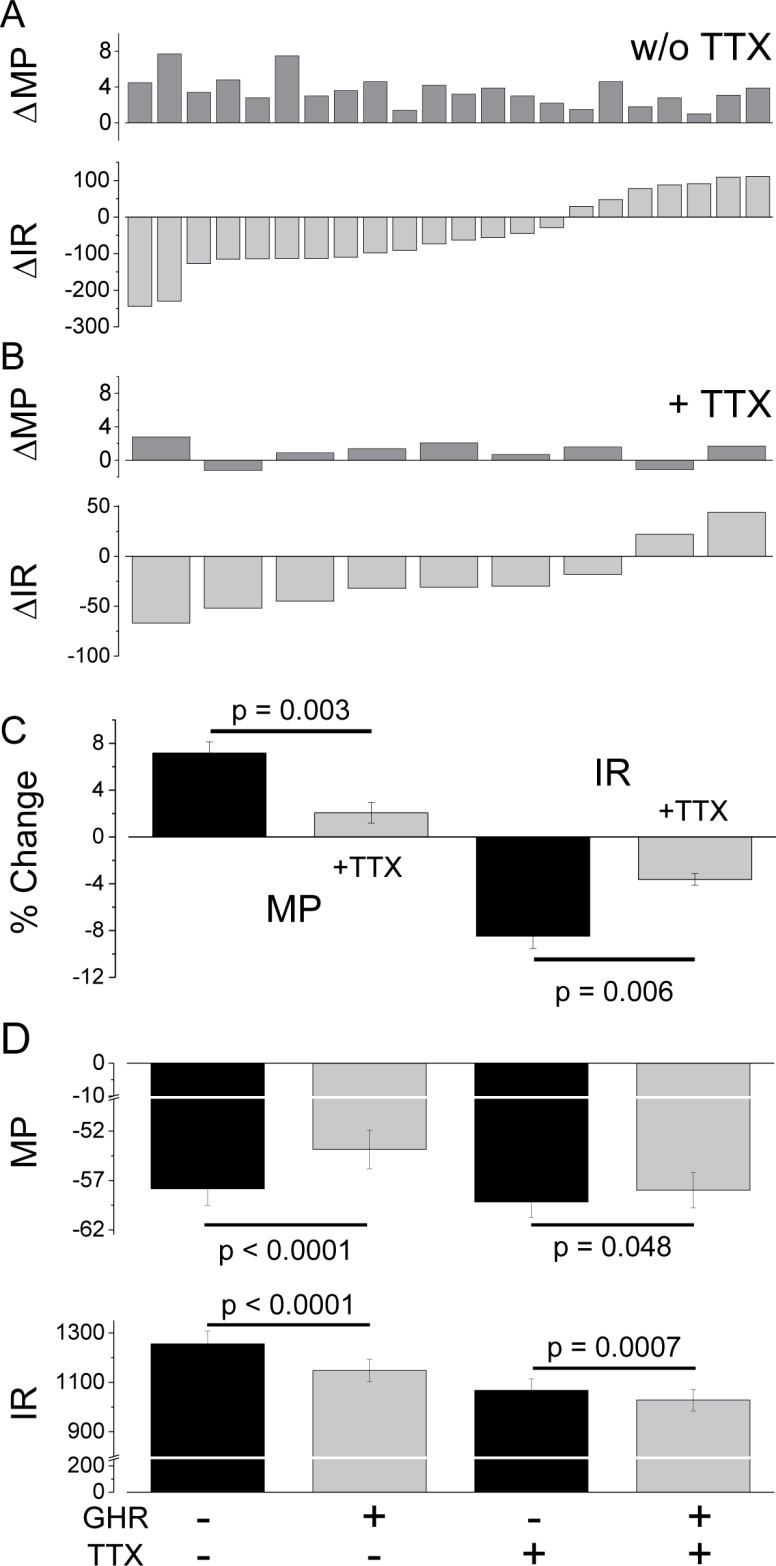
Effects of ghrelin (5 nM) on membrane potential (MP in mV) and input resistance (IR in MΩ) of NPY GFP neurons in VMH slices. The ΔMP (= MP^+ghrelin^ - MP^w/o ghrelin^) and ΔIR (= IR^+ghrelin^ - IR^w/o ghrelin^) values are plotted for individual neurons without (A) or with (B) added TTX (500 nM). (C) Unpaired, two-tailed t-test results for ghrelin-induced % Changes in MP and IR ± TTX in NPY-GFP neurons with decreased IR. (D) Paired, two-tailed t-test results indicate ghrelin can directly reduce the IR of NPY-GFP neurons when presynaptic inputs are blocked by TTX.

To assess the action(s) of ghrelin *in situ*, the MP and IR of green neurons in hypothalamic slices from NPY-GFP mice were determined as described [34], before and after ghrelin application, with or without added TTX. The difference in MP and IR values with or without ghrelin, i.e., ΔIR = IR^+Ghr^—IR^-Ghr^, are plotted for individual neurons. The MP values in Fig 1A show that application of ghrelin (5 nM) depolarizes all NPY-GFP neurons; average ΔMP value = 3.6 ± 1.7 mV (mean ± S.D). The IR data are consistent with depolarization in ~70% (15/22) of neurons due to decreases in IR, the result of opening cation channels, while the remainder (7/22) result from increases in IR presumably due to closing unidentified K^+^ or Cl^-^ channels. These differences are the sum of ghrelin effects on responsive presynaptic neurons and direct effects on NPY-GFP neurons. Comparison of Fig 1B with 1A shows that inhibiting presynaptic inputs with TTX reduces these ghrelin-induced changes in both MP and IR. Moreover, unpaired, two-tailed t-tests show that the changes in MP and IR in response to ghrelin in cells with decreased IR (average IR decrease = -108 ± 60 MΩ) was significantly reduced in the presence of TTX (MP, p = 0.003 and IR, p = 0.006; Fig 1C). These findings are consistent with a significant contribution from presynaptic inputs reported previously [35,36]. On the other hand, paired two-tailed t-tests comparing the MP and IR values in the presence and absence of ghrelin from neurons with decreased IR also show persistent, albeit diminished, ghrelin-induced changes in the presence of TTX. These significant effects in the presence of TTX (p = 0.048, MP and p = 0.0007, IR) are consistent with ghrelin directly affecting the activity of NPY-GFP neurons.

### Ghrelin directly activates isolated NPY-GFP neurons from the VMH

**Fig 2:**
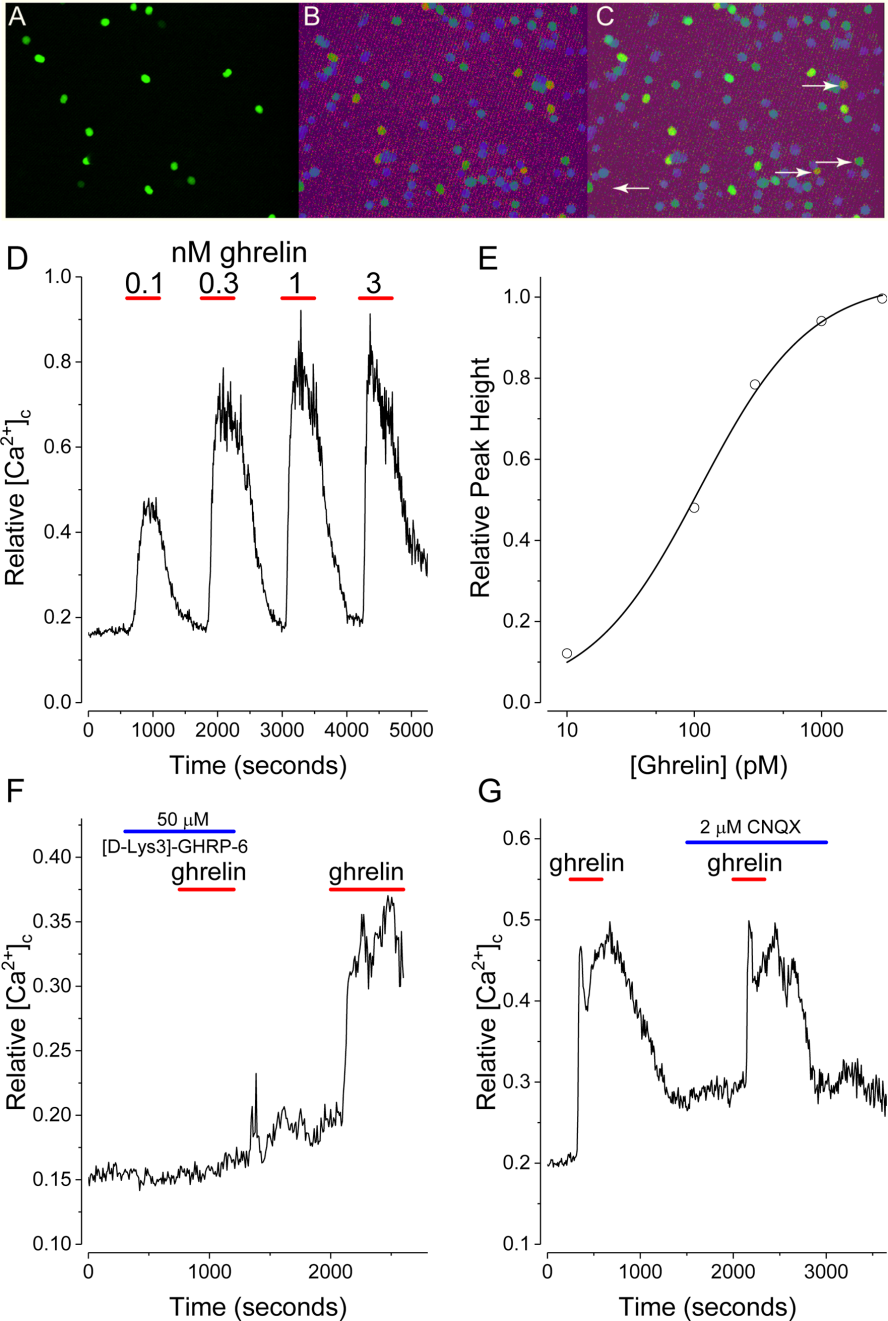
Ghrelin directly activates NPY-GFP neurons. (A) Representative field of isolated neurons from NPY-GFP mice. (B) Same field after addition of ghrelin. (C) Merged image of fields (A) and (B). Arrows point to non-NPY cells. (D) Average values (n = 4) of Relative [Ca^2+^]_c_ after sequential addition of indicated concentrations of acylated ghrelin. (E) Ghrelin dose-response curve. (F) Ghrelin induced [Ca^2+^]_c_ increases are blocked by the GHS-R antagonist [D-lys3]-GHRP-6 (50 μM). (G) CNQX (2 μM), an AMPA receptor antagonist, did not affect ghrelin action. Ghrelin (200 pM; red bars) was applied in F and G. Traces are mean values from 6–12 neurons.

To further assess direct effects of ghrelin, NPY-GFP neurons were studied after isolation. Fig 2A shows a representative field of ~100 cells, 16 of which show NPY-GFP green fluorescence. Fig 2B shows the same field ~6 minutes after addition of ghrelin (100 pM). In this field 16 of 16 NPY-GFP neurons plus 4 non-NPY cells were activated, as judged by elevated [Ca^2+^]_c_. The arrows in the merged image (Fig 2C) point to non-NPY neurons activated by ghrelin. Fig 2D shows the dose response of NPY-GFP neurons to ghrelin. In these experiments the [Ca^2+^]_c_ responses to applied ghrelin were measured in 6–12 NPY-GFP neurons. The EC_50_, determined from averages of the peak heights at a given ghrelin concentration, is approximately 110 pM (Fig 2E); 100 pM or 200 pM ghrelin was used for subsequent experiments. To confirm ghrelin acts through the growth hormone secretagogue receptor (GHS-R), Fig 2F shows that ghrelin action is blocked reversibly by [D-lys3]-GHRP-6, a GHS-R antagonist [1]. Since these neuron preparations are mixed cell cultures and presynaptic inputs are implicated in ghrelin action [35,36], the potential for NPY-GFP neuron activation by local release of glutamate was assessed. Fig 2G shows that the AMPA receptor antagonist, CNQX, had no significant effect on the action of ghrelin on isolated NPY-GFP neurons. While synaptic input is essential in a physiological setting, ghrelin activation in isolated cells was not the result of local release of glutamate. These experiments used agonist/antagonist perfusion times of ~10 minutes and were completely reversible. Continuous application of ghrelin for >30–40 minutes produced a sustained, but reversible [Ca^2+^]_c_ increase without apparent desensitization. Ghrelin washout and subsequent reperfusion produced essentially the same magnitude of [Ca^2+^]_c_ increase (data not shown).

### Ghrelin occupation of GHS-R in NPY neurons activates both PLC/IP_3_ and adenylate cyclase/cAMP/PKA signaling pathways

**Fig 3:**
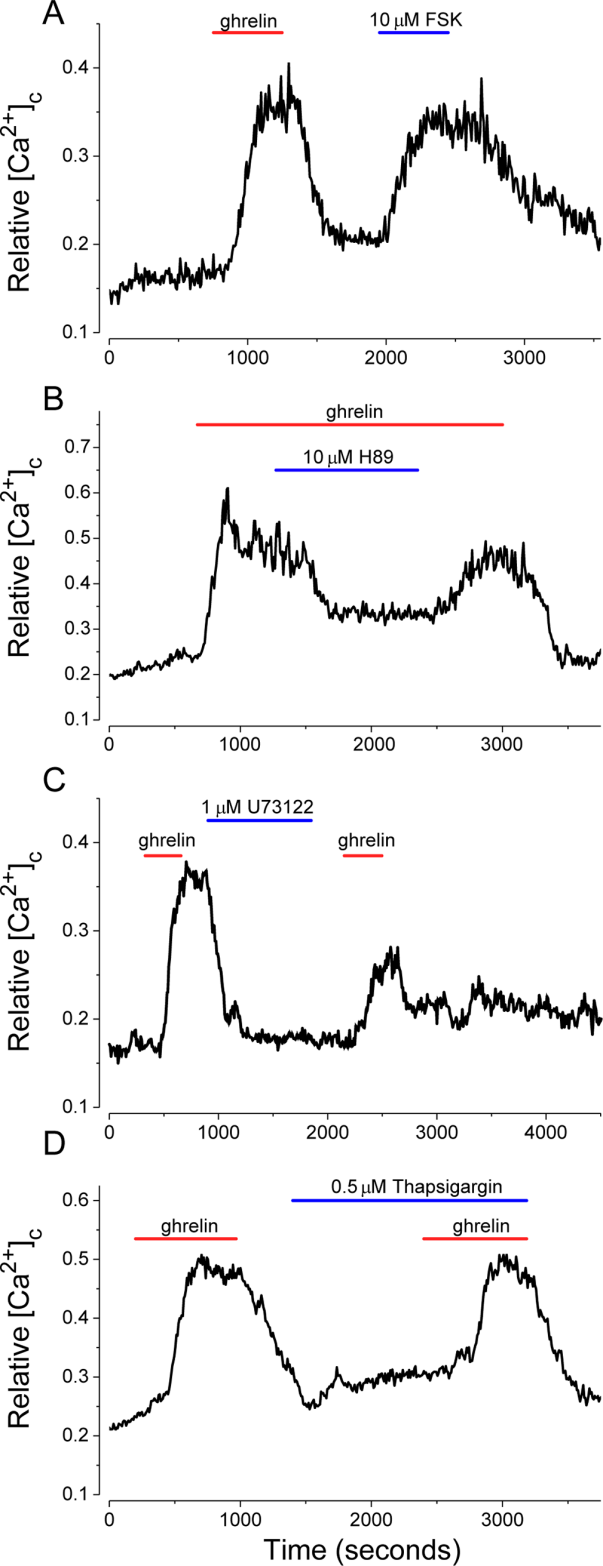
Ghrelin stimulation of GHS-R in isolated NPY-GFP neurons activates both adenylate cyclase (AC)-PKA and PLC-IP_3_ signaling pathways. (A) The adenylate cyclase agonist, Forskolin (10 μM), mimics the effect of ghrelin. (B) The PKA inhibitor, H89 (10 μM) inhibits the effect of ghrelin. (C) U73122 (1 μM), an irreversible PLC inhibitor, reduced ghrelin action. (D) Thapsigargin (0.5 μM), an inhibitor of SERCA, the ER Ca^2+^ pump, did not inhibit ghrelin induced [Ca^2+^]_c_ increases. Ghrelin was applied at 100 pM (red bars). Traces are mean values from 5–20 cells.

GHS-R acts through G-protein coupled receptors, Gs and Gq, to activate the adenylate cyclase/cAMP/PKA and PLC/IP_3_ pathways, respectively [9]. To assess whether these pathways are functional in isolated NPY-GFP neuron preparations, the effects of forskolin (FSK), an adenylate cyclase activator, and H-89, a PKA, inhibitor were tested. Activation of adenylate cyclase with FSK (10 μM) mimicked the action of ghrelin (100 pM, Fig 3A), while H-89 (10 μM) inhibits the stimulatory effect of ghrelin (100 pM) by >50% (Fig 3B). In equivalent experiments, both 5 μM H-89 and Rp-cAMP (20 μM), the latter a competitive inhibitor of PKA, inhibited ghrelin (100 pM) action by ~40% (data not shown). Fig 3C shows that a 15 minute preincubation with the irreversible PLC inhibitor, U73122 (1 μM), reduced subsequent ghrelin activation by >50%.

To assess the need for Ca^2+^ release from internal stores, thapsigargin was used to block endoplasmic reticulum Ca^2+^ pumps (SERCA) and empty internal stores. Thapsigargin (0.5 μM) elevated basal [Ca^2+^]_c_ somewhat, but did not block ghrelin action (Fig 3D). This result implied that ghrelin signaling through both the PLC/IP_3_ and adenylate cyclase/cAMP/PKA pathways was intact in these preparations, and that a significant amount of the observed ghrelin induced increase in [Ca^2+^]_c_ was due to influx of external Ca^2+^.

### Ca^2+^/calmodulin (CaM)-dependent protein kinase kinases (CaMKK2) and AMPK contribute to the ghrelin activation of NPY neurons

**Fig 4:**
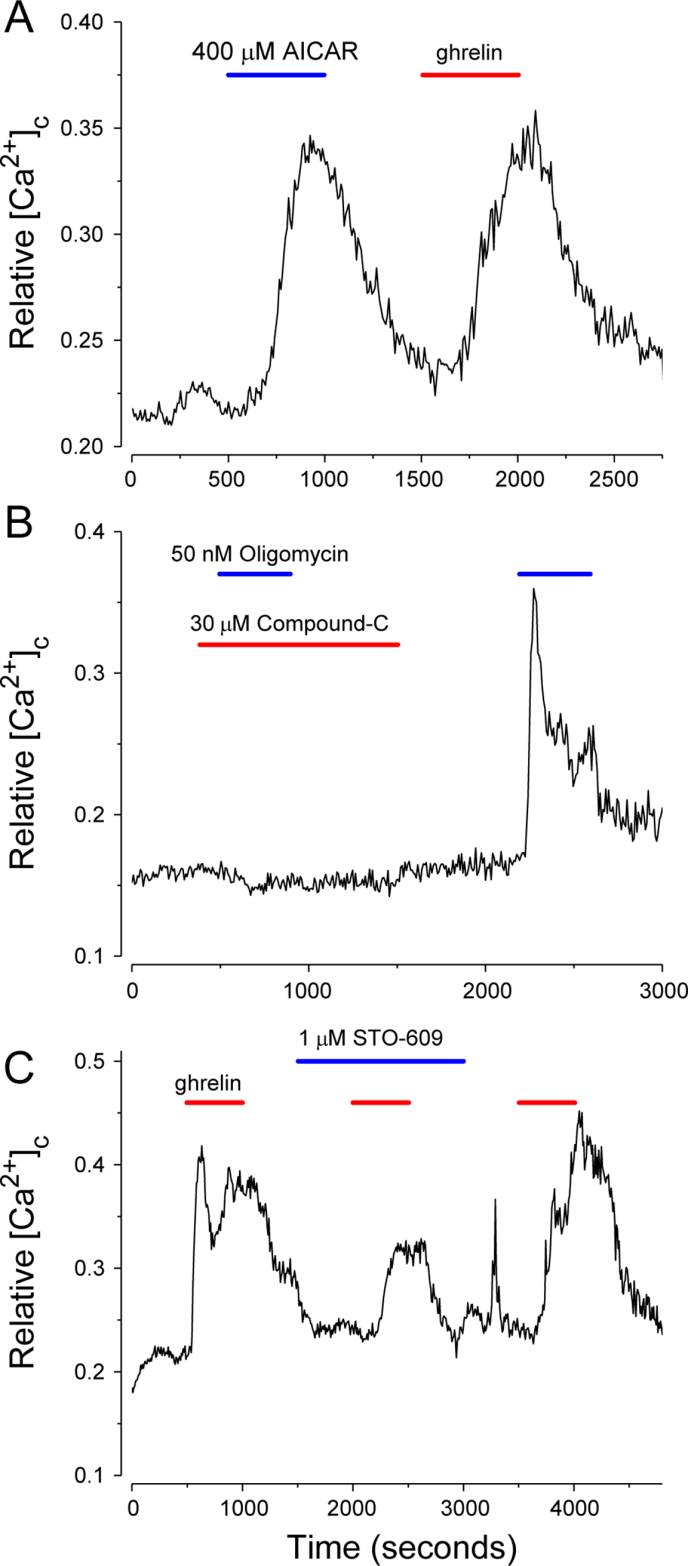
AMPK and Ca^2+^/calmodulin (CaM)-dependent protein kinase kinases (CaMKK2) contribute to the ghrelin activation of NPY neurons. (A) AICAR (400 μM), an AMPK activator, mimics the effect of ghrelin. (B) Compound-C (30 μM), an inhibitor of AMPK, completely blocks the effect of oligomycin. (C) STO-609 (1 μM), a CAMKK2 inhibitor, partially reverses the effect of ghrelin. Ghrelin was applied at 100 pM (red bars). Traces are mean values from 5–20 cells.

To confirm the functionality of AMPK signaling [37,38] in isolated neurons, the effects of the AMPK activator, aminoimidazole carboxamide ribonucleotide (AICAR) and the mitochondrial inhibitor oligomycin, used to shift energy balance in favor of an increase in intracellular AMP at the expense of ATP, were assessed. Stimulation of AMPK by AICAR (400 μM) produced the same effect on NPY-GFP neurons as 100 pM ghrelin (Fig 4A). Inhibition of oxidative phosphorylation by oligomycin (50 nM) increased [Ca^2+^]_c_, an action completely blocked by the AMPK inhibitor, compound C (Fig 4B). Compound C (30 μM) also markedly inhibited NPY-GFP neuron activation by ghrelin and FSK (data not shown). Fig 4C confirms that inhibition of CaMKK2 by STO-609 (1 μM), and thus phosphorylation of AMPK, partially blocks ghrelin activation [39]. The results confirm that AMPK activation and modulation by CaMKK2 are functional in our isolated NPY-GFP neurons.

### Gamma aminobutyric acid (GABA) modulates ghrelin action on NPY-GFP neurons

**Fig 5:**
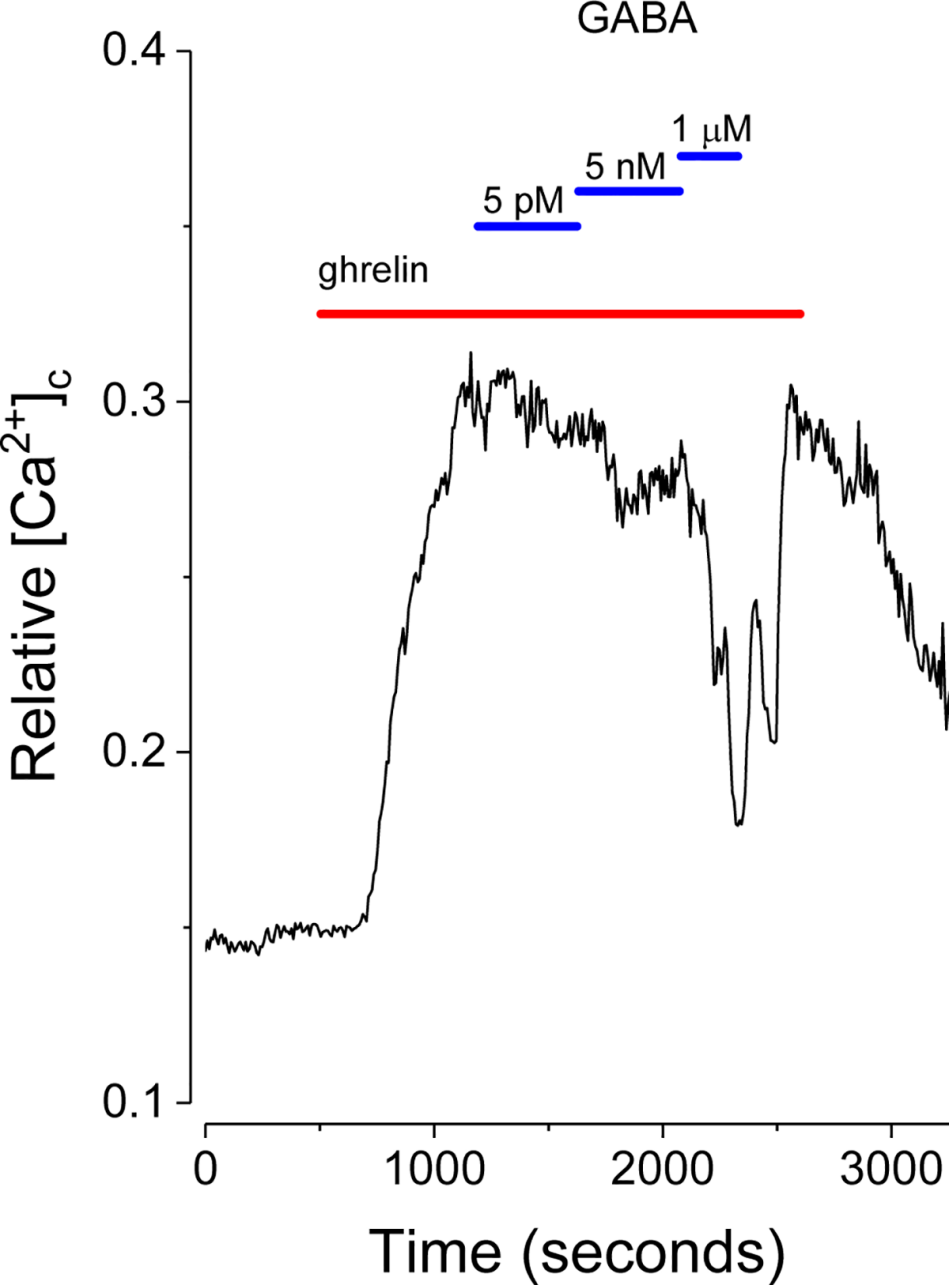
GABA inhibits the stimulatory effects of acylated ghrelin (100 pM) in a dose-dependent manner. The trace is the mean from 12 neurons.

Fig 5 confirms that NPY neurons are responsive to GABA [40]. The application of GABA (5 pM to 1 μM), to stimulate Cl^-^ currents through GABA_A_ channels, suppresses ghrelin activation in a dose-dependent fashion. The importance of Cl^-^ currents for setting the resting MP in NPY-GFP neurons was confirmed further by replacing extracellular NaCl with sodium gluconate. This substitution rapidly increased [Ca^2+^]_c_ consistent with the reduction of a hyperpolarizing inward Cl^-^ current (data not shown). The results are consistent with GABA-activated Cl^-^ currents acting tonically to suppress the action of ghrelin on NPY neurons in a physiologic setting and thus modulate orexigenic pathways.

### Mechanism(s) underlying Ca^2+^influx

**Fig 6:**
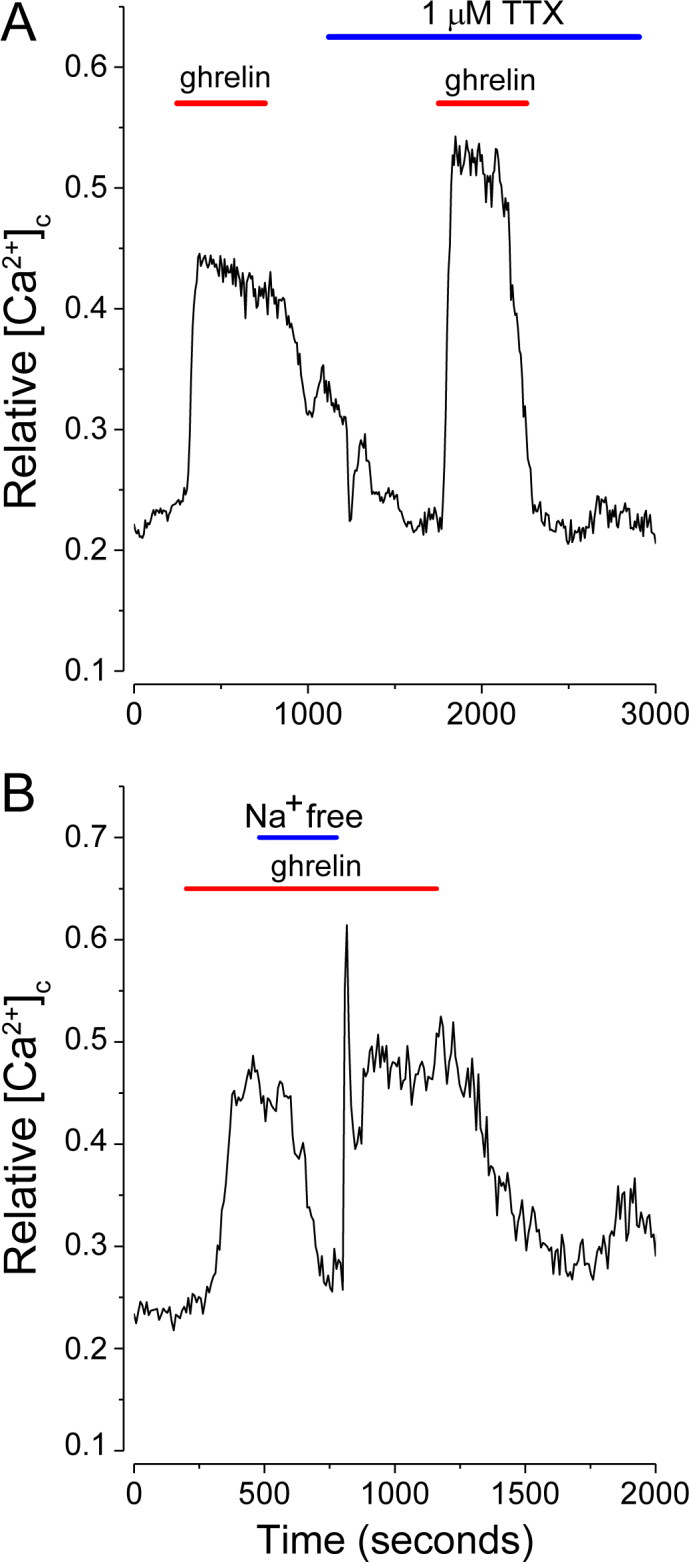
Extracellular Na^+^ influx is required to sustain ghrelin activation. (A) Tetrodotoxin (TTX; 1 μM), an inhibitor of voltage-dependent Na^+^ channels, did not affect ghrelin induced [Ca^2+^]_c_ increases. (B) The substitution of choline for Na^+^ rapidly and reversibly blocked the action of ghrelin. Ghrelin was applied at 200 pM (red bars). The traces are the mean values from 5–20 neurons.

While the signaling pathways downstream of GHS-R stimulation by ghrelin, confirmed above, are understood, the ion channels and Ca^2+^ entry pathways that underlie increased neuronal activity are less well defined. The experiment in Fig 3D, where thapsigargin was used to empty internal Ca^2+^stores, implies that maximal activation requires influx of extracellular Ca^2+^. To better define the relevant ionic pathways the roles of classic voltage-dependent Na^+^ channels, K_ATP_ channels, non-selective cation channels and voltage-gated Ca^2+^ channels were manipulated pharmacologically. Fig 6A shows that tetrodotoxin (1 μM), the classic inhibitor of voltage-dependent Na^+^ channels, has no effect on the action of ghrelin on isolated NPY-GFP neurons. The result is consistent with the lack of sensitivity to CNQX and absence of presynaptic inputs. The experiment in Fig 6B implies that extracellular Na^+^ influx is important and required to sustain ghrelin activation in this preparation. The substitution of Na^+^-free choline chloride for external NaCl rapidly and reversibly suppressed the action of 200 pM ghrelin.

**Fig 7:**
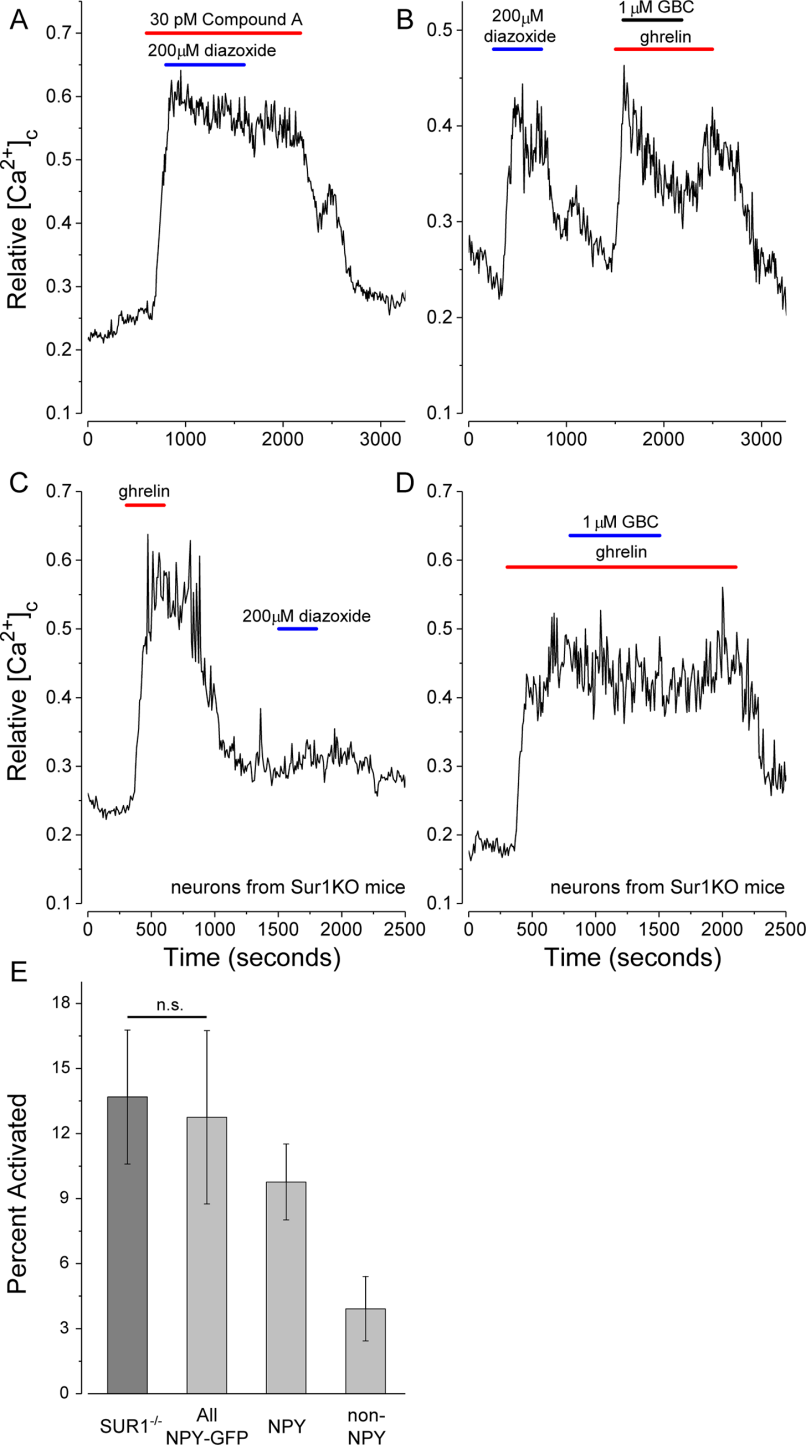
K_ATP_ channel modulators have SUR1 dependent effects on isolated NPY-GFP neurons. (A) Diazoxide (200 μM) did not affect ghrelin action. (B) When applied alone, diazoxide (200 μM) mimics the effect of ghrelin, while glibenclamide (1μM) reduced the stimulatory effect of ghrelin. (C-D) Neither diazoxide (200 μM) nor glibenclamide (1μM) affected ghrelin action in SUR1^-/-^ mice lacking SUR1. Ghrelin was applied at 200 pM as shown (red bars). (E) Comparison of the percentages of all ghrelin-activated cells in preparations of neurons isolated from SUR1^-/-^ versus NPY-GFP mice shows the mean values are not significantly different, p > 0.7, using an unpaired, two-tailed t-test. About two-thirds of the activated neurons in NPY-GFP mouse preparations were GFP-positive. Values are the means ± S.D. from four NPY-GFP and six SUR1^-/-^ preparations totaling more than 1000 cells.

SUR1/Kir6.2 type K_ATP_ channels have been identified previously in NPY neurons in the ARC and VMH [23,24,25]. To assess their potential role during ghrelin activation, the effects of glibenclamide and diazoxide, SUR1 selective antagonists and agonists, respectively, were tested. The expectation was that opening K_ATP_ channels with diazoxide would contribute a hyperpolarizing, outward K^+^ current, which would suppress ghrelin action similar to the effect of increased chloride (Cl^-^) influx induced by the application of GABA (Fig 5). Fig 7A shows that diazoxide (200 μM) has no inhibitory effect on NPY-GFP neurons activated by compound A, a GHS-R agonist, or by ghrelin (data not shown). Unexpectedly, the application of diazoxide alone reversibly activated the [Ca^2+^]_c_ response in all NPY-GFP neurons, while glibenclamide (1 μM) reversibly inhibited the action of ghrelin (Fig 7B). To assess whether SUR1 was required for this paradoxical effect, neurons were isolated from SUR1^-/-^ mice [41] using the same cell isolation protocol. Ghrelin (200 pM), but not diazoxide (200 μM), reversibly stimulated an increase in [Ca^2+^]_c_ in a subset of isolated SUR1^-/-^ neurons (Fig 7C) and glibenclamide (1 μM) had no significant effect on ghrelin action (Fig 7D) implying SUR1 is essential for the action of these channel modulators. The NPY neurons in SUR1^-/-^ mice are not GFP tagged. To reduce the likelihood that the ghrelin-activated neurons in the knockout animals represent a distinct subset, for example only the ghrelin-activated, non-NPY neurons shown in Fig 2A, the mean percentage of ghrelin-activated cells was determined in preparations from both NPY-GFP and SUR1^-/-^ mice. Fig 7E shows that the mean percentage of all activated neurons does not differ significantly between the two preparations. In the NPY-GFP preparations, approximately two-thirds of the activated neurons are GFP-positive. The results show SUR1 is essential for the paradoxical action of these channel modulators in NPY neurons, but also imply that SUR1 is not essential for activation by ghrelin.

**Fig 8:**
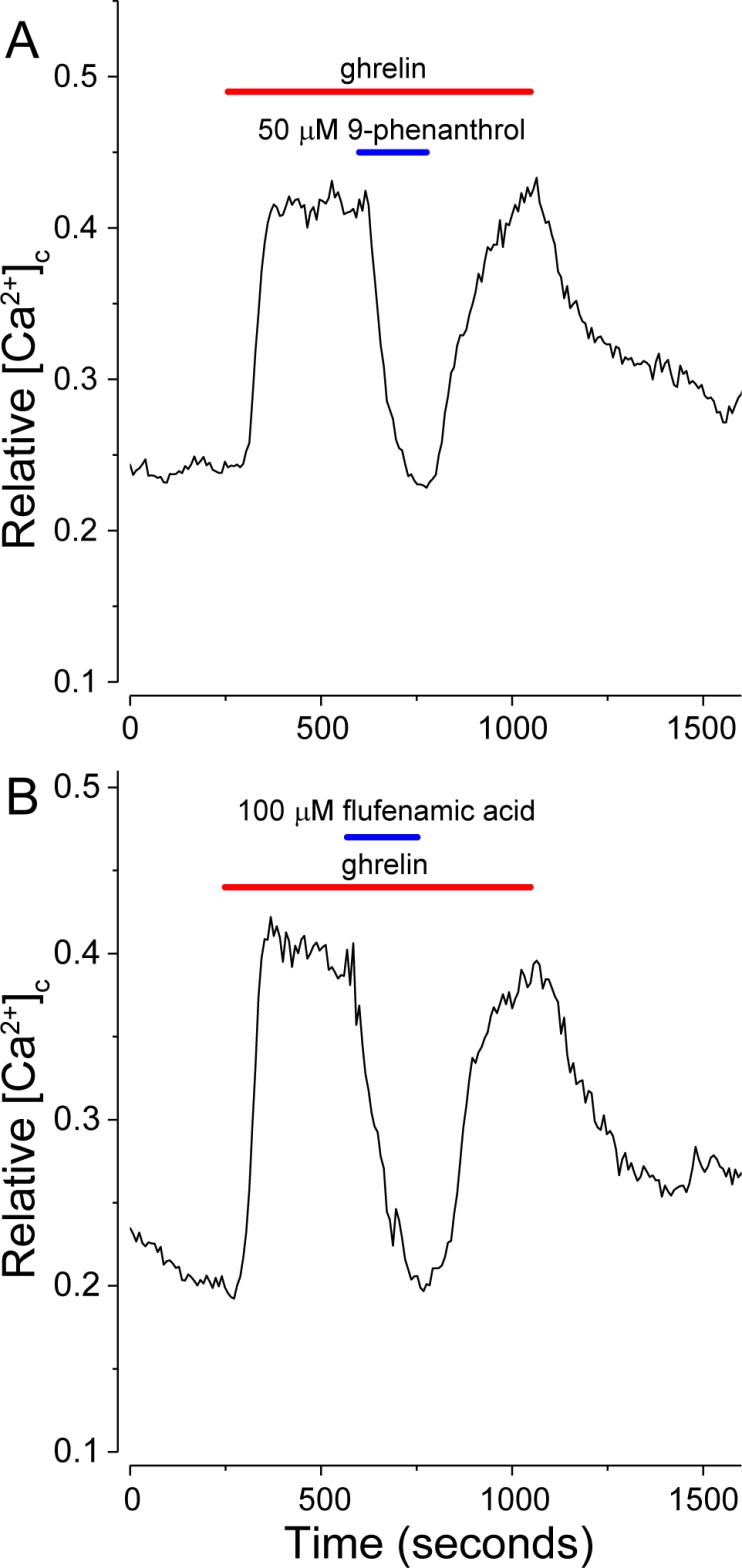
Antagonists of Trpm4 channels inhibit ghrelin action on isolated NPY-GFP neurons. (A) 9-phenanthrol (50 μM) and (B) flufenamic acid (100 μM) potently block the effects of ghrelin (200 pM; red bars).

The results in Fig 6 and in Fig 7 with SUR1 modulators suggest that SUR1 can couple with a depolarizing Na^+^ conductance, potentially the SUR1/Trpm4 non-selective cation channels described by Woo *et al*. [27], rather than with Kir6.2. In which case, diazoxide activation of SUR1 would stimulate an inward Na^+^ current and thus the observed depolarization. In support of this hypothesis two Trpm4 antagonists were tested. Fig 8A and 8B show that both 9-phenanthrol (50 μM), reportedly a selective inhibitor for Trpm4 versus Trpm5 channels [42], and flufenamic acid (100 μM), which potently inhibits recombinant Trpm4 channels expressed in HEK 293 cells [43], completely and reversibly block the action of 200 pM ghrelin on isolated NPY-GFP neurons. These results are consistent with the hypothesis that SUR1/Trpm4 channels are downstream effectors of ghrelin action in isolated NPY-GFP neurons and that a continuous influx of Na^+^ is needed for the sustained action of ghrelin seen in these preparations.

**Fig 9:**
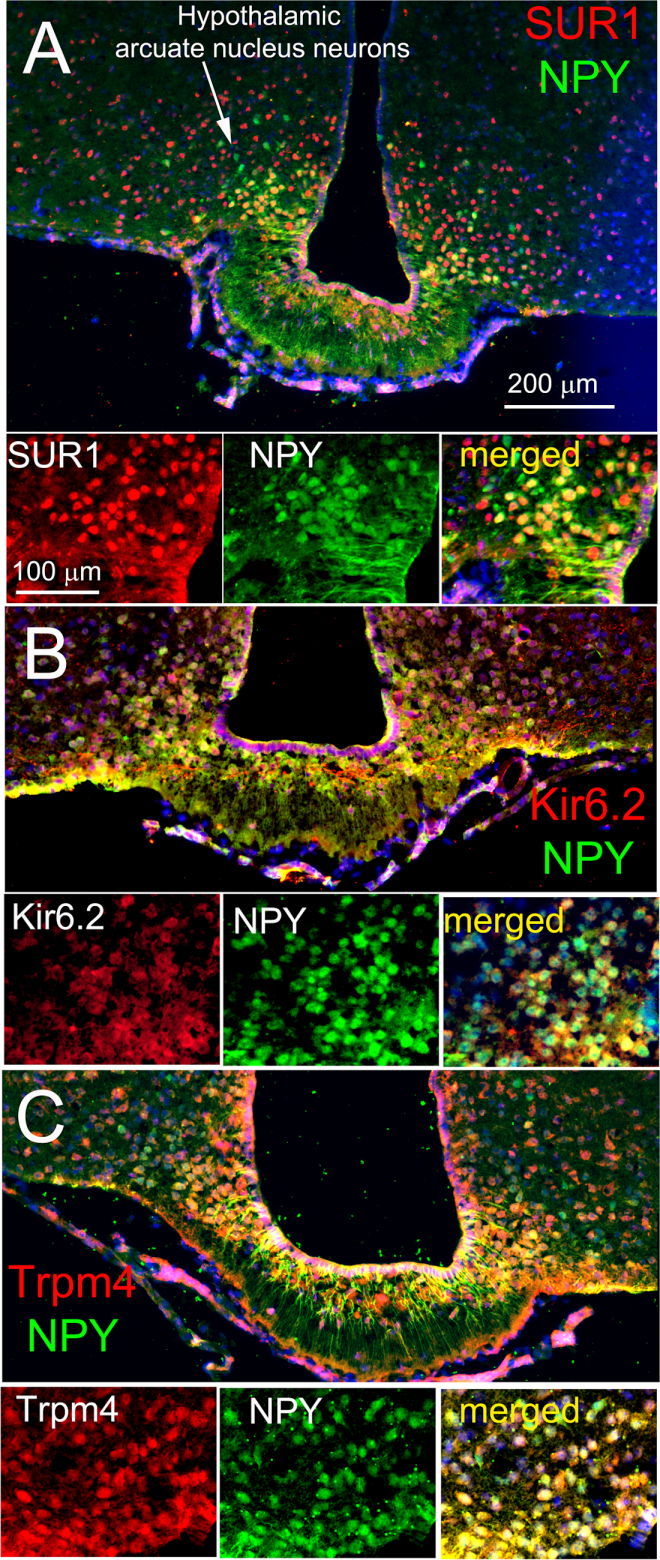
NYP-expressing neurons of the arcuate nucleus express subunits of both SUR1-Kir6.2 (K_ATP_) and SUR1-Trpm4 channels. Coronal sections of the hypothalamus from an NPY-GFP mouse, showing NPY-GFP neurons (green) immunolabeled for SUR1(A), or Kir6.2 (B) or Trpm4 (C), as indicated (red); merged images show co-expression of GFP and all three channel subunits by NPY neurons (yellow) DAPI nuclear labeling shown in blue. The images (low power view, *upper panel*s, and high power view, *lower panels*) shown are representative of findings in 2 NPY-GFP mice.

SUR1/Trpm4 channels [27] in the central nervous system have been studied extensively by Simard and colleagues (see [44,45,46] for review). First identified in reactive astrocytes [47,48] these non-selective cation channels are up-regulated in response to a variety of stimuli including traumatic neuronal injury where they exacerbate the pathologic outcome by contributing to cytotoxic edema and necrotic cell death (reviewed in [49,50]). Trpm4 and Trpm5 subunits have been identified in the hypothalamus, specifically in the magnocellular cells in the supraoptic nucleus and the paraventricular nucleus where they are implicated in the control of firing patterns of neurons expressing vasopressin and oxytocin [51]. Fig 9 shows that Trpm4, Kir6.2 and SUR1 are present in subpopulations of NPY neurons, and that individual neurons co-express both SUR1 and Kir6.2 as well as SUR1 and Trpm4. This is, to our knowledge, the first identification of Trpm4 channels in NPY neurons.

### Involvement of voltage-gated Ca2+ channels

**Fig 10:**
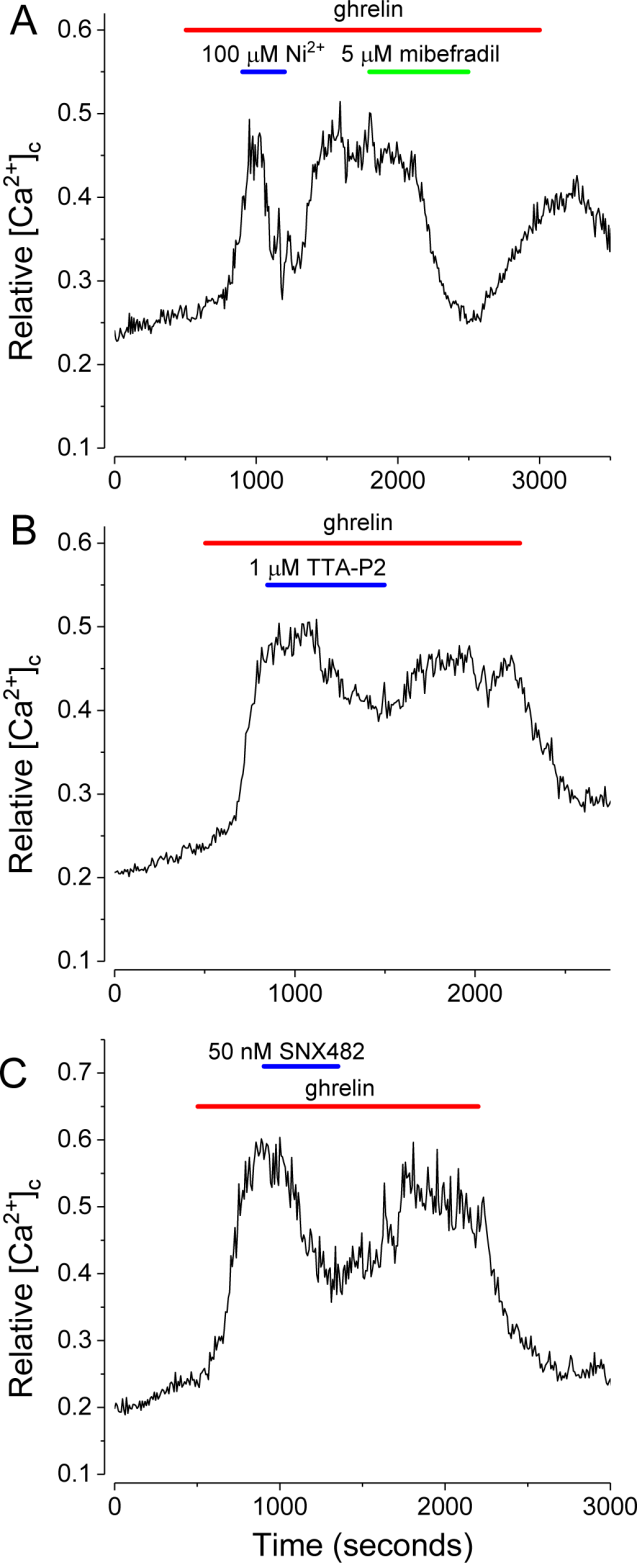
Blockade of T-type and R-type voltage dependent channels reduces the effect of ghrelin. (A) Ni^2+^ (100 μM), an inhibitor of T and R-type channels, and 5 μM mibefradil, an inhibitor of T and L type channels, inhibit the effect of ghrelin. (B) TTA-P2 (1 μM), a selective inhibitor of T-type channels, partially inhibited ghrelin action. (C) SNX482 (50 nM), an inhibitor of R-type channels, partially inhibits ghrelin induced [Ca^2+^]_c_ increases. Ghrelin was applied at 100 pM (red bars).

Trpm4 and/or SUR1/Trpm4 channels require Ca^2+^ for activity, but are impermeable to Ca^2+^ [52], thus the sustained elevation of [Ca^2+^]_c_ due to ghrelin is not the result of Ca^2+^ influx through these channels. To identify candidate Ca^2+^ channels, the effects of multiple voltage-gated Ca^2+^ channel antagonists were screened for their ability to suppress ghrelin action. Potent, selective inhibitors of L-type and N-type channels, nifedipine (10 μM; [53,54]) and ω-conotoxin (500 nM), respectively, had no discernible suppressive action on NPY-GFP neurons activated by 100 pM ghrelin (S1 Fig). Two channel inhibitors, Ni^2+^ (100 μM; [55]) and mibefradil (5 μM; [56,57], reported to semi-selectively block T-type channels, did markedly suppress ghrelin action (Fig 10A), while the more selective, higher affinity T-type antagonist, TTA-P2 (1 μM; [58]), partially inhibited ghrelin action (Fig 10B). A tarantula toxin, SNX-482, a high affinity antagonist reported to be selective for R-type Cav2.3 Ca^2+^ channels [59,60], potently inhibits ghrelin action at a concentration, 50 nM (Fig 10C). The potential inhibition of a K^+^ current [61] is not consistent with the observed repolarization.

## Discussion

The relative importance of ghrelin-modulated presynaptic inputs versus a direct ghrelin action on hypothalamic NPY neurons is not established. Early studies [62,63] proposed a direct action consistent with the activation of isolated NPY neurons by ghrelin [64,65], while recent studies emphasize the importance of ghrelin-responsive presynaptic neurons activated during food deprivation [35,36]. We confirmed that ghrelin depolarizes or activates NPY-GFP neurons in hypothalamic slices. Interestingly this response is quite heterogenous; depolarization resulted from graded decreases in IR in ~70% of NPY-GFP neurons and graded increases in the remainder (Fig 1). Consistent with earlier work [35], TTX blockade of presynaptic inputs to NPY neurons reduced ghrelin-induced changes in MP and IR significantly. However, although their responses were attenuated, TTX-treated NPY-GFP neurons were still significantly depolarized by ghrelin due to both decreases and increases in IR, although our data for the latter are too limited for analysis. These findings are consistent with both pre- and postsynaptic (direct) effects of ghrelin on NPY neurons. The identities of the ion channels that produce depolarization are not clearly established; we focus here on the channel(s) whose ghrelin-induced openings would *decrease* the IR and *depolarize* the MP.

To identify channels that contribute to the ghrelin-induced *decrease* in IR and MP depolarization of NPY neurons we used isolated hypothalamic NPY-GFP neuron preparations. To validate these preparations, earlier studies with isolated neurons were confirmed [64,65]. Specifically, the effects of ghrelin required GHS-R, were blocked by a ghrelin antagonist, required ~110 pM acylated ghrelin for half-maximal stimulation, and were not affected by CNQX, an AMPA receptor antagonist. In agreement with previous studies ghrelin occupation of GHS-R stimulated both adenylyl cyclase/cAMP/PKA and PLC/IP_3_ pathways; the action(s) of ghrelin on NPY-GFP neurons were stimulated by the adenylyl cyclase activator, forskolin, blocked by the PKA inhibitor, H89 and partially blocked by U73122, an irreversible PLC inhibitor. AMPK has been linked to ghrelin action [65,66,67] and these pathways activate AMPK in multiple ways, including via phosphorylation by CAMKII [39], thus we confirmed that the AMPK agonist, AICAR, produced a sustained rise in [Ca^2+^]_c_ in NPY-GFP neurons in the absence of ghrelin, that ‘compound-C’, a specific AMPK inhibitor and STO-609, a CAMKII inhibitor, reduced ghrelin action.

A role for Na^+^ fluxes in the action of ghrelin on isolated NPY-GFP neurons was shown by perfusing activated neurons with Na^+^ free medium and by using TTX, the classic Na^+^ channel blocker. TTX had no inhibitory effect, while the substitution of choline for Na^+^ rapidly and reversibly inhibited ghrelin action. These results implied that ghrelin-induced openings of an unspecified, TTX-insensitive Na^+^ conductance could underlie the *decreased* IR and MP depolarization observed in NPY-GFP neurons in TTX-treated hypothalamic slices. We suspected that this Na^+^ conductance could be due to the non-selective cation channel, Trpm4 ([68] reviewed in [69]), and potentially coupled with SUR1 to assemble the SUR1/Trpm4 channels described by Woo *et al*. [27]. This idea was tested by identifying SUR1, Kir6.2 and Trpm4 in hypothalamic NPY neurons, by assessing the effects of the SUR1 modulators, diazoxide and glibenclamide, on ghrelin actions in NPY-GFP neurons and by testing two Trpm4 inhibitors, 9-phenanthrol and flufenamic acid. We found that Trpm4, SUR1 and Kir6.2 co-localized immunohistochemically in NPY neurons in the arcuate nucleus. The SUR1 modulators are best known as ATP-sensitive K_ATP_ channel agonists and antagonists, respectively, where diazoxide-induced openings, for example in pancreatic ß-cells, result in MP *hyperpolarization* due to outward flux of K^+^. We observed, however, that diazoxide alone could activate NPY-GFP neurons, consistent with the idea that diazoxide agonism of SUR1 was opening SUR1/Trpm4 channels [27] to depolarize the cells, while glibenclamide antagonism of SUR1 inhibited depolarization. Both Trpm4 inhibitors rapidly and reversibly blocked ghrelin action. The measured mean MP of NPY-GFP neurons in 2.5 mM glucose in slice preparations was -57.3±5.5 mV (mean±S.D., n = 22). Thus, the MP of isolated neurons is expected to depolarize when SUR1/Trpm4 channels open. The combined results support the hypothesis that ghrelin can depolarize a subset of NPY-GFP neurons, i.e., those with a decrease in IR, by opening SUR1/Trpm4 non-selective cation channels.

The molecular link(s) between AMPK activation and channel openings in NPY neurons remains undefined. Modulation by phosphoinositides [70,71,72], activation by increased [Ca^2+^]_c_ levels [73] and phosphorylation by PKC [74] or Rho-associated kinase [75], reported to increase the affinity of Trpm4 for Ca^2+^, are all potential candidates by which AMPK activation could lead to Trpm4 channel opening (reviewed in [69]). We tested whether an interaction between SUR1 and Trpm4 is essential for ghrelin action using SUR1^-/-^ mice. The results show that while SUR1 is essential for the effects of diazoxide and glibenclamide, SUR1 is not required for ghrelin action. Ghrelin activated equivalent percentages of isolated neurons from SUR1^-/-^ and NPY-GFP mice (Fig 7E). What governs the partitioning of SUR1 between Kir6.2 and Trpm4 in cells expressing both is not understood, but the present results are consistent with the idea that AMPK, or a downstream effector, acts directly on Trpm4 which, in contrast to Kir6.2 [76,77], can traffic to the cell surface without SUR1 [78,79].

Ghrelin application produced a sustained increase in [Ca^2+^]_c_ which was stable for extended periods. Depletion of internal Ca^2+^ stores by thapsigargin did not block the sustained response, implying MP depolarization opens voltage-gated Ca^2+^ channels. In support of this mechanism, T- and R-type Ca^2+^ channel antagonists, but not L- or N-type inhibitors, partially blocked ghrelin action. This mechanism is distinct from a role in which opening Trpm4 channels reduces the driving force for Ca^2+^ entry through Ca^2+^ permeable Trp channels [68], for example in endothelial cells [80], mast cells [81] and microglial cells [82]. T-, but not R-type Ca^2+^ channels have been proposed to underlie sustained Ca^2+^ entry in other systems [83].

The activation of ARC NPY neurons during energy deprivation, for example during fasting, increases food intake, while it reduces energy expenditure [84,85,86,87] and increases hepatic glucose production [88,89]. Approximately 40% of NPY neurons, termed ‘GI’ neurons, in the ARC are inhibited by physiologic levels of extracellular glucose and activated when glucose levels fall [90,91]. The ionic mechanisms underlying the regulation of GI neurons are heterogeneous, but different than those described here for ghrelin. In an isolated VMH neuron preparation equivalent to ours, which is comprised of both ARC NPY-GI neurons as well as the much larger population of non NPY-GI neurons in the adjacent VMH, Murphy *et al*. [30] showed that ~15% of neurons were activated by low glucose via an AMPK-dependent mechanism. Unlike ghrelin activation, which *decreased* IR, in this mixed population of GI neurons AMPK activation stimulated the nNOS/NO/soluble guanylcyclase/cGMP pathway, whose upregulation *increased* IR and MP by closing a Cl^-^ channel, presumably CFTR *[30].* A distinct ionic mechanism not involving AMPK has been identified specifically for NPY-GI neurons using a more focused approach; Hao *et al*. [34] showed that ARC NPY-GFP neurons, presumably included in the isolated VMH and NPY-GFP populations, are activated directly by low glucose (0.1 mM vs 2.5 mM) by a mechanism in which increases in IR and MP are due to the closure of an unidentified K^+^ channel.

The upregulation of Trpm4 has been reported in multiple models of CNS injury and inflammation where the attenuation of the stimulatory effect of SUR1 on Trpm4 by glibenclamide or genetic knockout gives positive therapeutic outcomes [44,45,46]. Whether the activity of SUR1/Trpm4 channels in NPY neurons is modulated by pathologic, inflammatory conditions is not known, but it is worth pointing out that upregulation and/or increased activity of these channels is associated with neuroinflammation in CNS models [92,93,94], while inflammation within the hypothalamus is associated with obesity (e.g., [95,96,97,98]).

## Conclusions

NPY neurons have all the molecular machinery necessary to respond directly to ghrelin. Consistent with recent work, ghrelin modulates presynaptic inputs that activate NPY-GFP neurons directly *in situ*. Inhibition of presynaptic inputs shows that ghrelin can also directly depolarize neurons either by activating a depolarizing conductance(s) (decreased IR) or inhibiting a hyperpolarizing conductance(s) (increased IR). Results with isolated NPY-GFP neurons suggest the ghrelin-activated, depolarizing current is a Na^+^ conductance with the pharmacologic properties of SUR1/Trpm4 non-selective cation channels. In the isolated neuron model, the openings of SUR1/Trpm4 channels activate T- and SNX482-sensitive R-type voltage-dependent Ca^2+^ channels. During negative energy balance when ghrelin levels are elevated, openings of SUR1/Trpm4 channels could contribute a sustained depolarizing current to increase in NPY neuronal activity *in situ*.

## Supporting information

S1 Fig Inhibitors of L-type and N-type voltage dependent Ca^2+^ channels do not affect ghrelin action in isolated NPY-GFP neurons. (A) ω-conotoxin (500 nM), an inhibitor of N-type channels and (B) nifedipine (10 μM), an inhibitor of L-type channels, did not affect ghrelin induced [Ca^2+^]_c_ increases.
(TIF)
