# Supplementary figures and images for: Direct versus indirect actions of ghrelin on hypothalamic NPY neurons
Source: PLoS One. 2017 Sep 6;12(9):e0184261. doi: 10.1371/journal.pone.0184261 (PMC5587286; doi:10.1371/journal.pone.0184261)

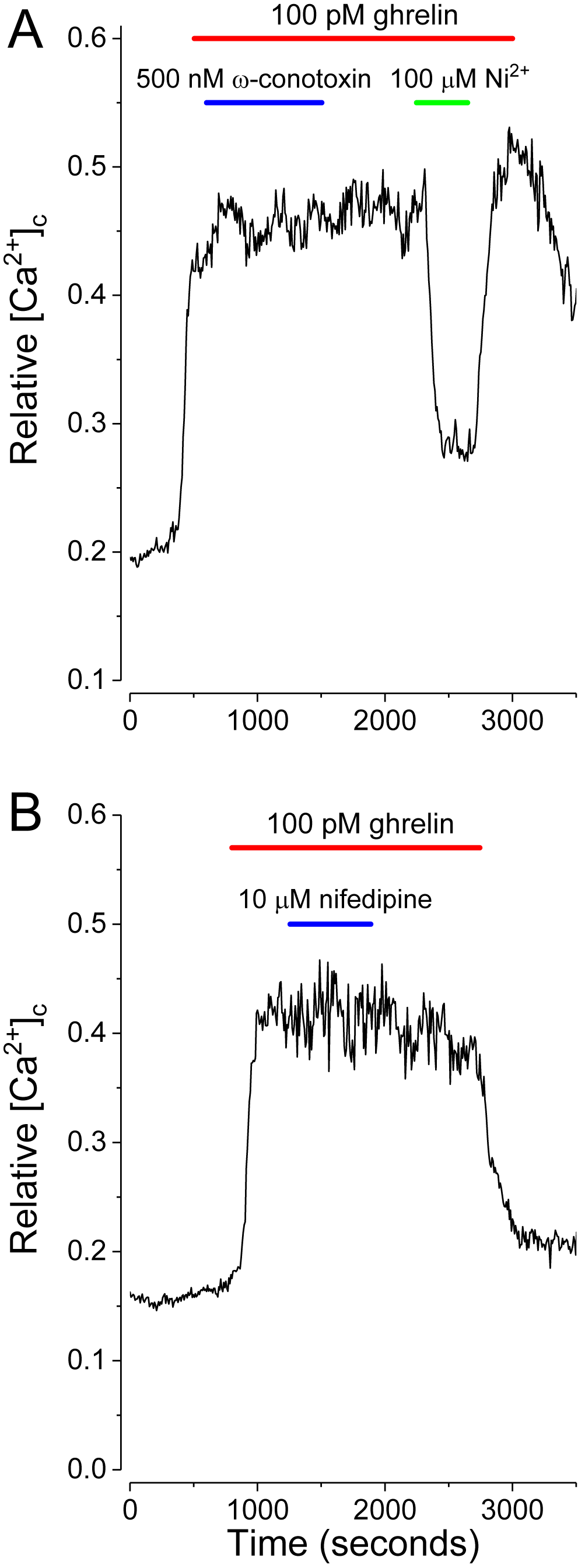

Supplement: S1 Fig — (A) ω-conotoxin (500 nM), an inhibitor of N-type channels and (B) nifedipine (10 μM), an inhibitor of L-type channels, did not affect ghrelin induced [Ca2+]c increases. (TIF) [file pone.0184261.s001.tif]
